# Antibacterial and Therapeutic Potentials of the *Capsicum annuum* Extract against Infected Wound in a Rat Model with Its Mechanisms of Antibacterial Action

**DOI:** 10.1155/2021/4303902

**Published:** 2021-10-04

**Authors:** Steve Endeguele Ekom, Jean-De-Dieu Tamokou, Victor Kuete

**Affiliations:** Research Unit of Microbiology and Antimicrobial Substances, Department of Biochemistry, University of Dschang, P.O. Box 67, Dschang, Cameroon

## Abstract

The wound healing process is essential to reform the damaged tissue and prevent its invasion by pathogens. The present study aims at evaluating the antibacterial and therapeutic properties of the *Capsicum annuum* L. (Solanaceae) extract against infected wound in a rat model with its mechanisms of antibacterial action. The fruit extract was prepared by maceration in methanol. The broth microdilution method was used to investigate the antibacterial activity of the methanol extract of *C. annuum* fruits. The therapeutic effect of the extract gel was performed on an excision wound infected with *Staphylococcus aureus* using a rat model. The total phenol, flavonoid, and tannin contents as well as the antibacterial mechanisms of action of the extract were determined using spectrophotometric methods. The *C. annuum* fruit extract showed antibacterial properties which can be linked to its total phenolic, flavonoid, and tannin contents. The antibacterial activity is due to the inhibition of the biofilm formation, ATPases/H^+^ proton pump, and dehydrogenase activity as well as the alteration of the bacterial cell membrane through the leakage of nucleic acids, reducing sugars and proteins. The extract gel showed a significant (*p* < 0.05) increase in the percentage of wound closure and eradicated *S. aureus* at the infection site. The extract gel was nonirritating to the skin and slightly irritating to the eyes and should be used with caution. Overall, the findings of the present study support the traditional use of the studied plant in the treatment of wounds and infectious diseases associated with the tested bacteria.

## 1. Introduction

Wounds are among the major and widely occurring pathologies [1]. The wound healing process is essential to reform the damaged tissue and prevent its invasion by pathogens [2]. Although wound healing is a fundamental process, the infection of wound can cause significant delays within the repair and regeneration cycle [3]. It has been reported that a key factor in delayed chronic wound repair was the failure of the host response to combat multifactorial infections including *Escherichia coli*, *Staphylococcus*, haemolytic *Streptococcus*, *Bacillus*, *Pseudomonas*, and *Proteus* species [4]. The understanding and control of the microbial infections are of great importance for the enhanced healing and management of wounds [5]. Continuous overuse of antibiotics has undoubtedly driven the development of their resistance. Inappropriate prescribing, extensive agricultural use, a decline in the availability of new antibiotics, and the various adaptations by which pathogenic bacteria obviate the effects of antimicrobials have further increased the threat [6]. As resistance to first- to fourth-generation antibiotics gains force, the development of new antimicrobial agents must be a priority if the problem is to be contained [7]. The plants are a large and unexplored reservoir of secondary metabolites, such as tannins, terpenoids, alkaloids, and flavonoids which have shown antimicrobial properties [8–11].

Pepper (*Capsicum annuum* L.) is a member of the *Solanaceae* family and is believed to be originated from Central and South America [12]. Peppers belong to the genus *Capsicum*. *C. annuum*, *C. frutescens*, *C. chinense*, *C. pubescens*, and *C. baccatum* are grown domestically or commercially [13]. Peppers are excellent sources of phytochemicals, such as anthocyanins, vitamins, phenolic acids, flavonoids, carotenoids, and capsaicinoids [14, 15]. Numerous studies have demonstrated the antibacterial activity of capsaicin and dihydrocapsaicin bioactive compounds isolated from *Capsicum annuum* [16, 17]. *C. annuum* fruits are traditionally used in wound healing, but no scientific evidence is found in the literature that supports its activity. Furthermore, no report on its mode of action has been seen in the literature. Hence, to justify its traditional use in Cameroon, the present study is aimed at evaluating the antibacterial and therapeutic activities of the methanol extract of *C. annuum* fruits (MECa) against infected wound in the rat model and unraveling its mechanisms of antibacterial action.

## 2. Materials and Methods

### 2.1. Plant Collection and Extract Preparation

Dried fruits of *Capsicum annuum* were purchased in January 2019 at a market in Foumbot, situated in the western region of Cameroon. Identification and authentication of the plant were done at the Cameroon National Herbarium, where the voucher specimen was kept under the reference number 25872/HNC. The dried fruits were powdered in an electric blender. The preparation of the extract was carried out as previously described [18]. Briefly, 300 g of the powder was macerated in 2 L of methanol for 48 h at room temperature with occasional shaking. The mixture was filtered using Whatman no. 1. The filtrate obtained was concentrated using a rotary evaporator at 65°C and placed at 40°C in an oven to eliminate the remaining solvent. The resulting extract was kept at 4°C until use.

### 2.2. Bacterial Species and Culture Media

The antibacterial activities of the methanol extract were carried out against three strains and four isolates of bacteria (Table 1). The clinical isolates of *Staphylococcus aureus* and *Escherichia* coli were taken from the Research Unit of Microbiology and Antimicrobial Substances (RUMAS) collection (Cameroon). The bacterial characteristics are summarized in Table 1. The tested bacteria were maintained at 4°C on Mueller–Hinton agar (MHA) [19, 20]. Mueller–Hinton broth (MHB) was used for the determinations of minimum inhibitory concentrations (MICs) and minimum bactericidal concentrations (MBCs).

### 2.3. In Vitro Antibacterial Assay

INT colorimetric assay [21, 22] was performed to assess the minimum inhibitory concentrations (MICs) of MECa against a panel of 7 Gram-negative and Gram-positive bacteria (Table 1). Briefly, plant extract was first dissolved in dimethyl sulfoxide (DMSO). The solution obtained was then added to Mueller–Hinton broth (MHB) and serially diluted twofold (in a 96-well microplate). One hundred microliters (100 *μ*L) of inoculum (1.5 × 10^6^ CFU/mL) prepared in MHB was added. The plates were covered and agitated to mix the contents of the wells using a plate shaker and incubated at 37°C for 24 h. The final concentration of DMSO was 1% and does not affect the microbial growth. Wells containing MHB, 100 *μ*L of inoculum, and DMSO at a final concentration of 1% served as a negative control whereas wells containing amoxicillin trihydrate, cefotaxime, levofloxacin, and erythromycin were used as positive controls. The MIC values of samples were determined by adding 40 *μ*L of a 0.2 mg/mL *p*-iodonitrotetrazolium violet solution followed by incubation at 37°C for 30 min. Viable microorganisms reduced the colorless dye to pink. MIC was defined as the lowest sample concentrations that prevented this change and exhibited complete inhibition of bacterial growth. All assays were performed in triplicate and repeated three times. For the determination of MBC values, a portion of liquid (5 *μ*L) from each well that showed no growth of bacteria was plated on Mueller–Hinton agar and incubated at 37°C for 24 h. The lowest concentrations that yielded no growth after this subculturing were taken as the MBC values.

### 2.4. Mode of Antibacterial Action of the MeOH Extract from *C. annuum* Fruit (MECa)

#### 2.4.1. Outer Membrane Permeability to Erythromycin

The potentiating effect of MECa on the membrane permeability to erythromycin was performed as previously described [23] with slight modifications. Briefly, three bacterial species were selected: *S. aureus* 18, *P. aeruginosa* PA01, and *E. coli* 64R. 50 *μ*L of Mueller–Hinton Broth was introduced into each well. Then, 50 *μ*L of erythromycin solution (20 *μ*g/mL) was added and microdiluted to concentrations of 0.07 to 10 *μ*g/mL. 50 *μ*L of the stock solution of extract 4096 *μ*g/mL was added to the MIC in each well. Each well contained a unique combination of plant extract and varying antibiotic concentrations. Then, 50 *μ*L of bacterial inoculum prepared as described above (10^6^ CFU/mL) was added. The whole was incubated at 37°C for 24 h on a shaker. For each concentration of extract to be tested, we had available 3 test wells (extract + erythromycin), 3 test wells (extract alone), and 3 test wells (erythromycin alone). After 24 hours of incubation, bacterial growth was measured in a microplate reader at 450 nm. The test was performed in triplicate.

#### 2.4.2. Loss of 260 nm Absorbing Material

Nucleic acid leakage has been used as an indicator of major damage to the cytoplasmic membrane. The effect of MECa on the nucleic acid leakage through the bacterial cell membrane was performed as earlier described [23] with slight modifications. The bacterial cultures of *S. aureus* 18, *P. aeruginosa* PA01, and *E. coli* 64R in the logarithmic growth phase in Mueller–Hinton Broth were centrifuged at 3500 rpm for 30 min, then washed, and resuspended in 10 mM PBS (pH 7.4). Bacterial strains were incubated with extract at MIC for different time periods (3, 6, 9, and 12 h). Bacterial strains incubated with PBS alone served as a control. The mixture was filtered through a 0.2 *μ*m membrane to remove bacterial cells, and the absorbance of the filtrate was determined in a spectrophotometer (Biobase Bk-D590 Double Beam Scanning UV/Vis, China) at 260 nm. The test was carried out in triplicate.

#### 2.4.3. Loss of Proteins and Reducing Sugars

In order to determine the leakage of reducing sugars and proteins through the bacterial membrane, a volume of 15 mL of culture containing 10^8^ CFU/mL of bacteria was incubated at 37°C for 24 hours. The resulting culture was centrifuged at 3500 × g for 10 min; the pellet obtained was washed with physiological water and suspended in the same water. Then, a volume of the bacterial suspension of *S. aureus* 18, *P. aeruginosa* PA01, and *E. coli* 64R and a volume of the extract prepared with distilled water were introduced into tubes so as to have MIC of extract in each tube. A negative control (distilled water + suspension) was made. The whole was incubated at 37 ± 2°C and stirred on a shaker at 150 rpm for 24 h. After incubation, 1 mL of each sample was taken and centrifuged at 12000 rpm for 30 min (at 4°C) and the supernatant was collected and immediately frozen at −20°C. The concentrations of reducing sugars and proteins were determined using a spectrophotometer (Biobase Bk-D590 Double Beam Scanning UV/Vis, China) at 540 and 595 nm, respectively [24, 25].

#### 2.4.4. Assay on the Enzymatic Activity of Respiratory Chain Dehydrogenases in the Bacteria

The dehydrogenase activity was evaluated according to the previous iodonitrotetrazolium chloride method [26, 27]. Under physiological conditions, colorless INT is reduced by the bacterial respiratory chain dehydrogenase to a dark-red water-insoluble iodonitrotetrazolium formazan (INF); thus, the dehydrogenase activity can be determined by the change of the spectrophotometric value of INF. Briefly, different volumes of MHB medium bacterial culture were added separately in 5 mL of cultures which gave final concentrations of extract corresponding to the MIC of extract except on the isolate *E. coli* 64R where it was 1/4 MIC of extract and final concentrations of 10^8^ CFU/mL for the different bacterial isolate. Amoxicillin was used as a control. A negative control containing cultures of three bacterial isolates *S. aureus* 18, *P. aeruginosa* PA01, and *E. coli* 64R were boiled for 20 min to completely inactivate the enzymes, while the unboiled cultures whose enzymes maintained native activity were considered as the control (+). Cultures were performed at 37°C with constant shaking at 150 rpm. One milliliter of culture was sampled and centrifuged separately at 10000 rpm for 30 min, then, the supernatants were discarded, and the bacteria were washed with phosphate-buffered saline (PBS) twice; then, 0.9 mL PBS was added to suspend the bacteria. INT solution (0.1 mL 0.5%) was added and the culture was incubated at 37°C in the dark for 2 h; then, 50 *μ*L of formaldehyde was added to stop the reaction. The culture was centrifuged to collect the bacteria, and 250 *μ*L of acetone and ethanol 1 : 1 (*v*/*v*) were used to distill the INF twice. The resulting supernatants were finally collected, and the dehydrogenase activity was then calculated based on the maximum spectrophotometrical absorbance of INF at 490 nm by a spectrophotometer (Biobase Bk-D590 Double Beam Scanning UV/Vis).

#### 2.4.5. Evaluation of the Effect of Methanol Extract on the H^+^/ATPase-Mediated Proton Pumps

The proton pump activity is mediated by acidification of the external environment of bacteria [28]. 500 *μ*L of bacterial suspension of *S. aureus* 18, *P. aeruginosa* PA01, and *E. coli* 64R obtained from an 18 h bacterial culture was introduced into MHB so as to obtain a volume of 50 mL of bacterial culture and then incubated at 37°C for 18 hours. The resulting culture was centrifuged at 3500 × g for 10 min, and the pellet was washed with distilled water and then with 50 mM KCl and resuspended in 50 mL of 50 mM KCl. The suspension was then stored at 4°C for 18 hours for glucose deprivation, and the pH was adjusted to 6.4 by adding HCl and/or NaOH. In 4 mL of this solution, 0.5 mL of extract solution dissolved in 3% DMSO and distilled water was added to obtain the concentrations of the extract equal to the MIC concentrations except on the isolate *E. coli* 64R where it was 1/4 MIC. After 10 minutes of preincubation at 37°C, the acidification of the medium was initiated by adding 0.5 mL of 20% glucose solution, the rapid catabolism of which will be accompanied by the release of protons in the medium. The pH of the medium was measured every 20 minutes for 1 hour. For this test, amoxicillin was used as positive control. The noted pH values made it possible to draw curves of variation of the pH as a function of time. Inhibition of the acidification of the medium in the presence of the extract was attributed to an inhibitory effect on the functioning of ATPase/H^+^ proton pumps by the extract.

#### 2.4.6. Antibiofilm Assay

The ability of MECa to inhibit biofilms was determined according to the 3-[4,5-dimethylthiazol-2-yl]-2,5 diphenyl tetrazolium bromide (MTT) colorimetric method [29]. Briefly, 100 *μ*L of MHB supplemented with 2% glucose was introduced into a 96-well microplate. The extract was introduced into the upper wells and microdiluted in a geometric progression of order 2. Then, 100 *μ*L of inoculum prepared with 0.5 McFarland bacterial suspension of *S. aureus* 18, *P. aeruginosa* PA01, and *E. coli* 64R was introduced into the wells. Controls represented by the growth control (MHB and inoculum), positive control (inoculum and amoxicillin), and sterility control (MHB alone) were also done. The plates were incubated at 37°C for 48 hours. After incubation, the microplates were washed with 50 mM phosphate-buffered saline (pH 7.4, 25 Mm NaCl) and were subsequently dried. The biofilm formed by the adherent cells was fixed with 100 *μ*L of MTT solution (5 mg/mL) and incubated at 37°C for 2 hours. The wells of the plate were subsequently emptied; then, 200 *μ*L of 10% DMSO was added. Optical densities were measured using a microplate reader at 570 nm. There is inhibition of the biofilms if the amount of biofilm formed decreases compared to that of the control group untreated with the extract. The percent inhibition was calculated using the formula:

(1) $$\begin{array}{c} \%\text{of inhibition}=\frac{\text{OD of the growth control}-\text{OD of the test}}{\text{OD of the growth control }}\times 100. \end{array}$$

### 2.5. Wound Healing Assay

#### 2.5.1. Experimental Animals

For this study, sixty-three (63) male *Wistar* albino rats aged 8 to 10 weeks and weighing 150 to 200 g were used. The breeding took place in the animal house of the Department of Biochemistry, University of Dschang, Cameroon. The rats were housed in individual polypropylene cages at 23 ± 1°C in 12 h : 12 h, dark : light cycle. The animals received standard diet and water *ad libitum* and were starved 12 h before the beginning of the experiment. Efforts were also made to minimize animal suffering and to reduce the number of animals used in the experiment.

#### 2.5.2. Preparation of the Gels

The gels were prepared by gently dispensing a calculated mass of polymer (Carbopol 940) into a known volume of water in order to obtain 1% Carbopol. The mixture was kept under constant stirring using a magnetic stirrer at 800 rpm for 1 hour to avoid lump formation in the dispersion. In the course of stirring, the mixture was neutralized by dropwise addition of triethanolamine (TEA) solution and stirring continued until a clear viscous homogeneous gel was obtained. The pH was adjusted to 7.5, and the mixture was kept for a period of 24 h for complete swelling and equilibration of the polymer. Finally, the extract solutions prepared at different concentrations (1%, 5%, and 10% *w*/*w*) using distilled water were added to the previous mixture with uninterrupted stirring till the extract totally dispersed in the hydrogel. After mixing the 1% Carbopol base with the different concentrations of extract, the pH was evaluated. The final formulations were packed in small-mouth plastic containers covered with plastic lid and kept at 24°C until use. Four types of gel were prepared: the gel containing only 1% Carbopol and those containing 1%, 5%, and 10% of *C. annuum* fruit extract.

#### 2.5.3. Creation and Contamination of Excision Wound

The wound site was prepared following the excision wound model as previously described [18] with some slight modifications. The rats were anesthetized before and during the infliction of experimental injuries with a dose of ketamine (50 mg/kg body wt, IM), and surgeries were performed under sterile conditions. The dorsal fur of the rats was shaved with an electric clipper and the delimitation of the wound site was made using a marker and then disinfected with 70% alcohol. The excision was performed by cutting the skin with a diameter of 2 cm and a depth of 1 to 2 mm thickness at the level of the depilated area along the marking using a scalpel and a sharp pair of scissors. The wound was inoculated with 150 *μ*L of overnight *S. aureus* 18 inoculum (10^8^ CFU/mL) and left undressed. Each animal was placed isolated in a disinfected cage in the disinfected animal facility in the Department of Biochemistry, Faculty of Science, University of Dschang. To verify the establishment of the infection, the isolation and identification of *S. aureus* from the infected area were done by culturing scabs taken from the infection site in petri dishes containing Mannitol salt agar medium. Treatment started 24 h after the establishment of the infection by dermal application of the extract gel once per day for 20 consecutive days till complete epithelization. Animals were randomly assigned into nine groups of seven animals per group. One group was without wounds, not infected, and not treated (unwounded group). One group was noninfected and untreated (uninfected group). Infected rats were divided into seven groups of seven animals each (four control groups and three test groups). The first control group was not treated (untreated group), and the second, third, and fourth control groups received gel without extract (blank group), clindamycin (Aclin gel), and Baneocin® (2%) 250 UI/5000 UI (Baneocin group), respectively. All three test groups were each treated topically with 1, 5, and 10% (*w*/*w*) of gel based on *C. annuum* fruit extract. The efficacy of the treatment was evaluated on a clinical and mycological basis. Clinical efficacy was based on changes observed at the site of infection during the test.

#### 2.5.4. Assessment of the Clinical Efficacy

The gel was topically applied on each wound once a day until complete epithelization. Assessment of the clinical efficacy was done by monitoring the wound contraction and wound closure time. The diameter of excised wounds was measured with the aid of a millimeter rule on days 4, 8, 12, 16, and 20 postinjury.

Wound contraction (%) was calculated as percentage reduction in the wound area using the following formula [30]:

(2) $$\begin{array}{c} \text{Wound contraction }\left(\%\right)=\frac{{\text{WA}}_{0}-{\text{WA}}_{t}}{{\text{WA}}_{0}}x100, \end{array}$$

where WA_0_ is the wound area on day zero and WA_*t*_ is the wound area on day *t*.

#### 2.5.5. Mycological Evaluation

For the mycological efficacy, animals were anesthetized with chloroform vapors at the end of the treatment (20^th^ day) and a skin specimen from the wound area (4 g) was cut out and ground in a porcelain mortar in the presence of 4 mL of physiological saline (NaCl, 0.9%) and the ground product obtained was centrifuged at 3000 rpm for 15 min. The supernatant obtained after centrifugation was decanted and used for culture on Mannitol salt agar medium in order to count the number of colony-forming units (CFU) of *Staphylococcus aureus* per gram of skin.

#### 2.5.6. Blood Collection

Before starting the treatment (on the 3^rd^ day), two rats of each group were euthanized by an overdose of ketamine and the blood was collected from the tail. At the end of the treatment (on the 20^th^ day), blood samples were collected by cardiac puncture from ketamine-overdosed rats into nonheparinized tubes. The tubes were allowed to stand 1 hour in ice and centrifuged at 3500 rpm for 10 min to obtain the serum. The serum obtained was used to assay total protein concentration and lactate dehydrogenase (LDH) enzyme activity using commercial kits: BIOLABO, L.D.H. (LDH-P), SBFC Modified Method, REF 92011, and Chrono Lab, Total protein, Cat. no. 101-0240.

#### 2.5.7. Skin Irritation Test

The skin irritation test with a gel based on *C. annuum* fruit extract was performed using a rat model as previously described [18, 31] with some slight modifications. Five rats were used for each gel concentration, and their skins were shaved on the dorsal side, each about a 500 mm^2^ area 24 h prior to application of the gel. The experimental animal received a single dose of the gel on the skin, and an area of the untreated skin was used as a control. 500 mg of *C. annuum* extract gel was applied uniformly to a shaved area of the skin, and this area was covered with an adhesive tape. Reactions related to the test gel were observed 1, 24, 48, and 72 h post application [32]. The presence of edema, erythema, and pressure sores in the treated skin was observed, and the skin reactions were assessed by grades of skin irritation.

#### 2.5.8. Eye Irritation Test

The eye irritation test was performed as earlier described [18]. Five rats were used for each gel concentration. The animals were immobilized and placed individually in a compression box where 100 mg of the gel at different concentrations of *C. annuum* fruit extract was instilled into the conjunctival sac of one of the animal's eyes after discarding the hair from the eyelids. The untreated eye was used as a control. Observations of the ocular for irritation were done 1, 24, and 48 h post instillation of the gel [33]. The lesions of the eye were assessed according to the nature and severity of the lesions and whether or not they were reversible and numerically by scores.

#### 2.5.9. Characterization of Gels

1. *Organoleptic Control*

The gel was kept for a period of 24 h for complete swelling and equilibration of the polymer prior for the organoleptic control. This was done by evaluating the odor, color, and appearance of the gel over storage time [34].

(2) *Control of Homogeneity*

The control of homogeneity was carried out by spreading the gel in order to check for the presence or absence of lumps and air bubbles.

#### 2.5.10. Physicochemical Test

1. *Determination of pH*

It was done by measuring the pH of the gel using a pH meter 24 h after the gel preparation and over storage time (28 days).

### 2.6. Chemical Analysis of the MeOH Extract of *C. annuum* Fruits

The presence of different classes of compounds (alkaloids, flavonoids, coumarins, anthraquinones, tannins, saponins, anthocyanins, triterpenes, phenols, polyphenols, and sterols) was detected in MECa using standard methods [35]. Also, the total phenol, flavonoid, and tannin contents were determined by the spectrophotometric methods [36–38]. The gallic acid, catechin, and tannic acid solutions prepared at different concentrations were used to construct the standard curves in order to determine the concentrations of total phenols, flavonoids, and tannins, respectively, in the extract.

### 2.7. Statistical Analysis

The data were expressed as mean ± standard deviation (SD). Statistical analysis was performed using one-way analysis of variance (ANOVA) with post hoc Waller-Duncan's multiple range tests with SPSS 23.0 for Windows. *p* < 0.05 was considered significant.

## 3. Results

### 3.1. Antibacterial Activity of the MeOH Extract of *C. annuum* Fruits

The antibacterial activity of the plant extract was evaluated by determining the minimum inhibitory concentrations (MICs) and minimum bactericidal concentrations (MBCs) against Gram-positive and Gram-negative bacteria (Table 2). The MeOH extract of *C. annuum* fruits inhibited the growth of all the tested bacteria (MIC = 64–2048 *μ*g/mL). The lowest MIC value of 64 *μ*g/mL was recorded against *S. aureus* 18 whereas the highest MIC value of 2048 was obtained on *E. coli* 64R. This finding suggests that *S. aureus* 18 and *E. coli* 64R were the most sensitive and resistant bacteria, respectively, to the methanol extract. However, the MIC values of MECa on *S. aureus* 18 are higher than those obtained with cefotaxime, levofloxacin, and erythromycin used as standard antibiotics. Interestingly, *S. aureus* 18 was found resistant to amoxicillin (MIC > 16 *μ*g/mL) while *E. coli* 64R was resistant to amoxicillin, cefotaxime, and erythromycin (MIC ≥ 16 *μ*g/mL). The antibacterial activity of the methanol extract against *E. coli* ATCC 8739 was higher than that of amoxicillin and equal to that of erythromycin. Apart from *E. coli* 64R and *E. coli* 141R, the MBC values were recorded on the sensitive bacteria and were larger than their corresponding MIC values.

### 3.2. Mode of Antibacterial Action of the MeOH Extract of *C. annuum* Fruits

#### 3.2.1. Effect of the MeOH Extract of *C. annuum* Fruits on the Outer Membrane Permeability

**Figure 1:**
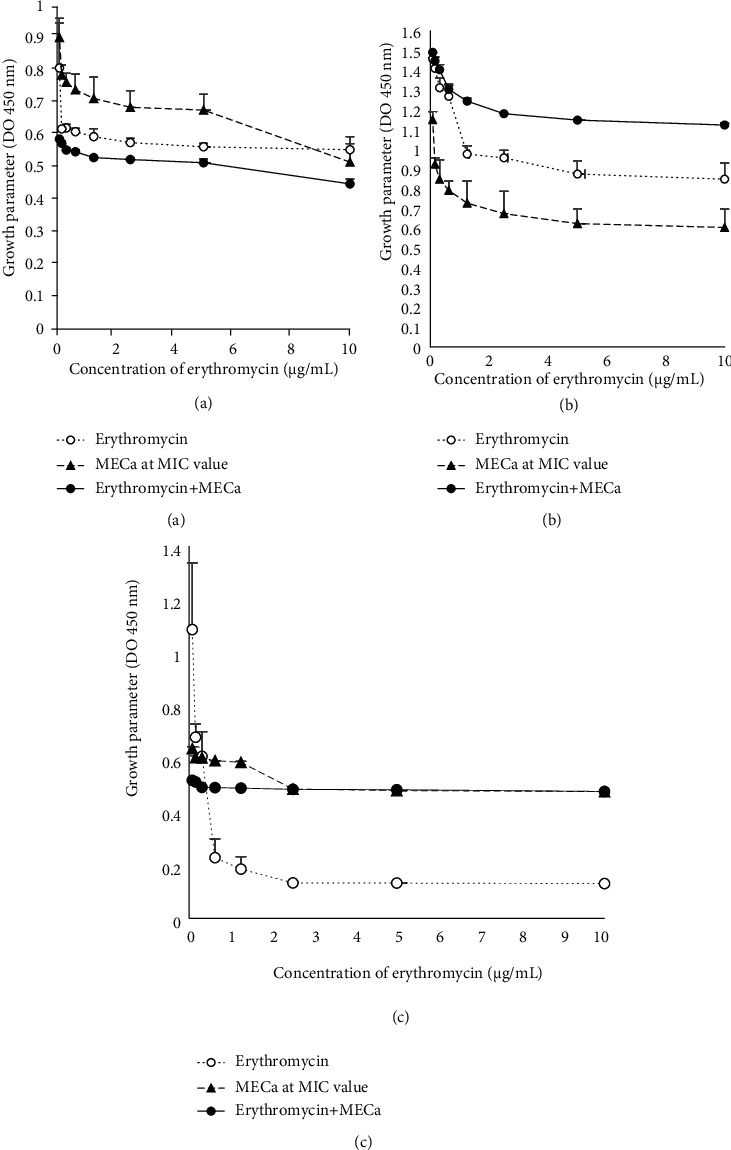
Bacterial outer membrane permeability to erythromycin induced by the MeOH extract of *C. annuum* fruits (MECa). Each value represents mean ± SD of three independent assays; (a) *Pseudomonas aeruginosa* PA01; (b) *Staphylococcus aureus* 18; (c) *Escherichia coli* 64R.

The coincubation of MECa with bacterial suspension had measurable inhibitory effect on the bacterial growth just as erythromycin that caused bacterial growth inhibition (Figure 1). The combination of the MECa with erythromycin caused *Pseudomonas aeruginosa* PA01 to be more susceptible to the erythromycin at all the tested concentrations (Figure 1(a)). The potentiating effect of the MECa on the membrane permeability to erythromycin was not observed against *E. coli* 64R and *S. aureus* 18 where the effect of the erythromycin + MECa was lesser than that of the erythromycin alone (Figures 1(b) and 1(c)). Meanwhile, the effect of the MECa alone on the outer membrane permeability was higher than that of the combination on *S. aureus* 18 at all the tested concentrations (Figure 1(b)).

#### 3.2.2. Effect of the MeOH Extract on the 260-Nm-Absorbing Material

**Figure 2:**
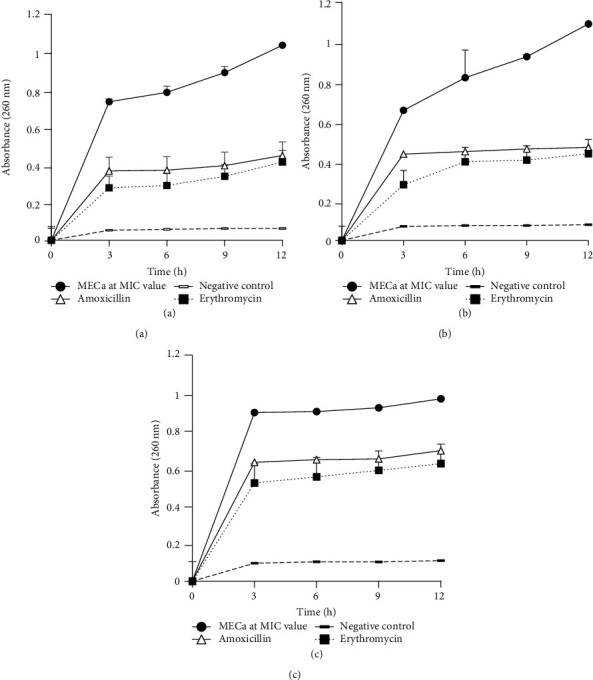
Nucleotide leakage from bacterial suspensions treated with MIC of MeOH extract of *C. annuum* fruits (MECa). Each value represents mean ± SD of three independent assays; (a) *Pseudomonas aeruginosa* PA01; (b) *Staphylococcus aureus* 18; (c) *Escherichia coli* 64R.

The MECa caused significant increases in the OD_260_s in all bacterial suspensions. This was observed from the third hour of incubation with a maximum effect at 12 hours when compared to the respective negative control culture (Figure 2). The effects of MECa were also greater than those of the amoxicillin and erythromycin used as standard controls.

#### 3.2.3. Effect of the MeOH Extract on the Reducing Sugar and Protein Leakages

**Figure 3:**
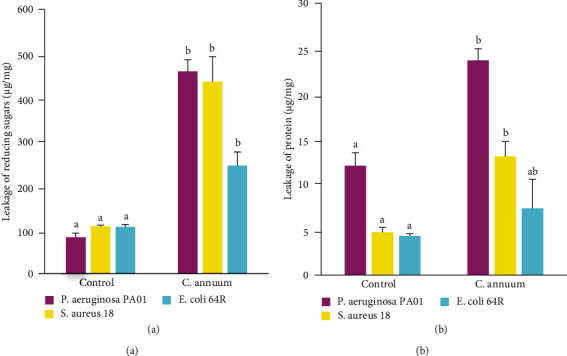
Leakage of reducing sugars (a) and proteins (b) from bacterial suspension treated with the MeOH extract of *C. annuum* fruits. Each value represents mean ± SD of three independent assays; values affected by the different superscript letters (a, b) are significantly different (*p* < 0.05).

The leakages of reducing sugars and proteins were significantly (*p* < 0.05) highest in the bacterial suspensions treated with the MeOH extract when compared to the untreated cells (Figure 3). This result suggests that the MeOH extract accelerates the escape of reducing sugars and proteins from the bacterial cytoplasm. Furthermore, the MeOH extract induced the highest leakages of reducing sugars (Figure 3(a)) and proteins (Figure 3(b)) in *Pseudomonas aeruginosa* suspension following in decreasing order by those of *S. aureus* 18 and *E. coli* 64R.

#### 3.2.4. Effect of the MeOH Extract on the Enzymatic Activity of Respiratory Chain Dehydrogenases in the Bacteria

**Figure 4:**
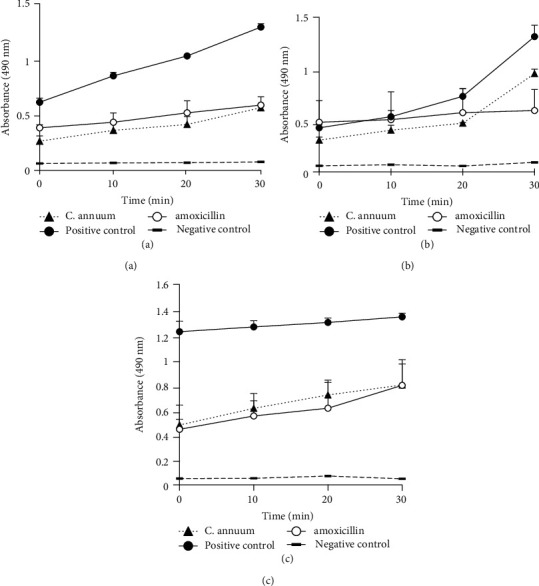
Effect of the MeOH extract of *C. annuum* fruits on the enzymatic activity of respiratory chain dehydrogenases. Data are average from triplicate experiments. Error bars represent standard deviations of triplicate incubations. The negative and positive controls represent the boiled and unboiled bacteria, respectively. (a) *Pseudomonas aeruginosa* PA01; (b) *Staphylococcus aureus* 18; (c) *Escherichia coli* 64R.

Irrespective of the incubation times, the enzymatic activities of respiratory chain dehydrogenases in the bacterial suspensions treated with the MeOH extract were lower than those of the positive controls (Figure 4). Interestingly, at incubation times lower or equal to 20 min, the dehydrogenase activities in the *Pseudomonas aeruginosa* and *Staphylococcus aureus* suspensions treated with the MeOH extract were lower than those of amoxicillin. However, after 30 min of incubation, the dehydrogenase activities of *P. aeruginosa* and *S. aureus* suspensions treated with the MeOH extract were equal to and higher than those of amoxicillin, respectively (Figures 4(a) and 4(b)). Furthermore, the dehydrogenase activities of *E*. *coli* 64R suspension treated with the MeOH extract were in general greater than those of amoxicillin (Figure 4(c)). Our results also show an increase in the dehydrogenase activity of positive control (unboiled cells) over time of incubation whereas the dehydrogenase activity showed virtually no change in the negative control (boiled cells).

#### 3.2.5. Effect of the MeOH Extract on the ATPases/H^+^ Proton Pumps

**Figure 5:**
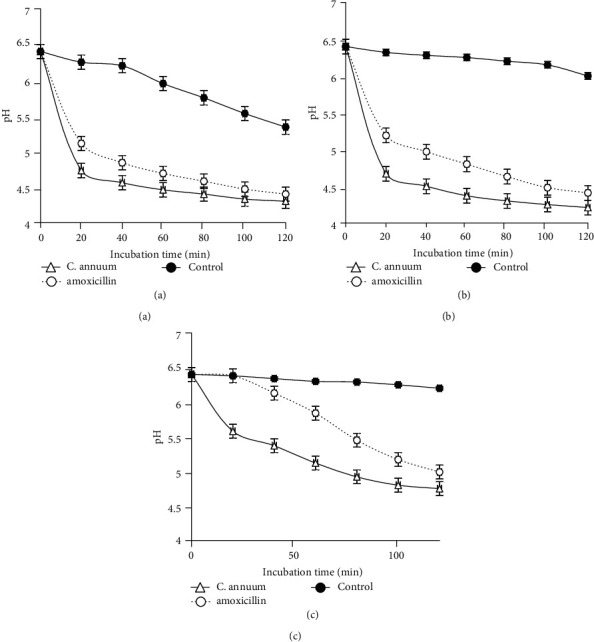
Effect of the MeOH extract of *C. annuum* fruits on the bacterial ATPase/H^+^ proton pumps. Data are average from triplicate experiments. Error bars represent standard deviations of triplicate incubations. (a) *Pseudomonas aeruginosa* PA01; (b) *Staphylococcus aureus* 18; (c) *Escherichia coli* 64R.

The MECa at its MIC value showed ATPases/H^+^ proton pump inhibition effects. This inhibitory effect was more pronounced on *Pseudomonas aeruginosa* PA01 and *Staphylococcus aureus* 18 (Figures 5(a) and 5(b)).

#### 3.2.6. Antibiofilm Activity of *C. annuum* Fruit on the Selected Bacteria

**Figure 6:**
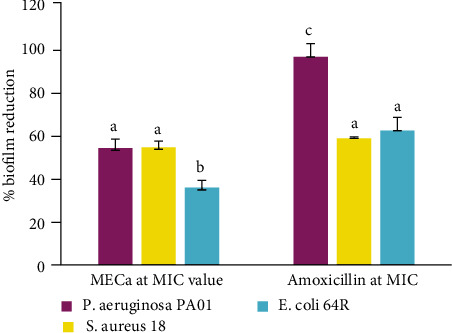
Antibiofilm effect of the MeOH extract of *C. annuum*fruits against the tested bacteria. Data are expressed as mean ± SD. *n* = 3. Values with different letters are significantly (*p* < 0.05) different according to the Waller-Duncan test.

The antibiofilm potential of the MECa was significantly (*p* < 0.05) lesser than that of amoxicillin against *P. aeruginosa* and *E. coli* (Figure 6). However, the antibiofilm activity of the MeOH extract was comparable to that of amoxicillin against *S. aureus*.

### 3.3. In Vivo Antibacterial Activity

**Figure 7:**
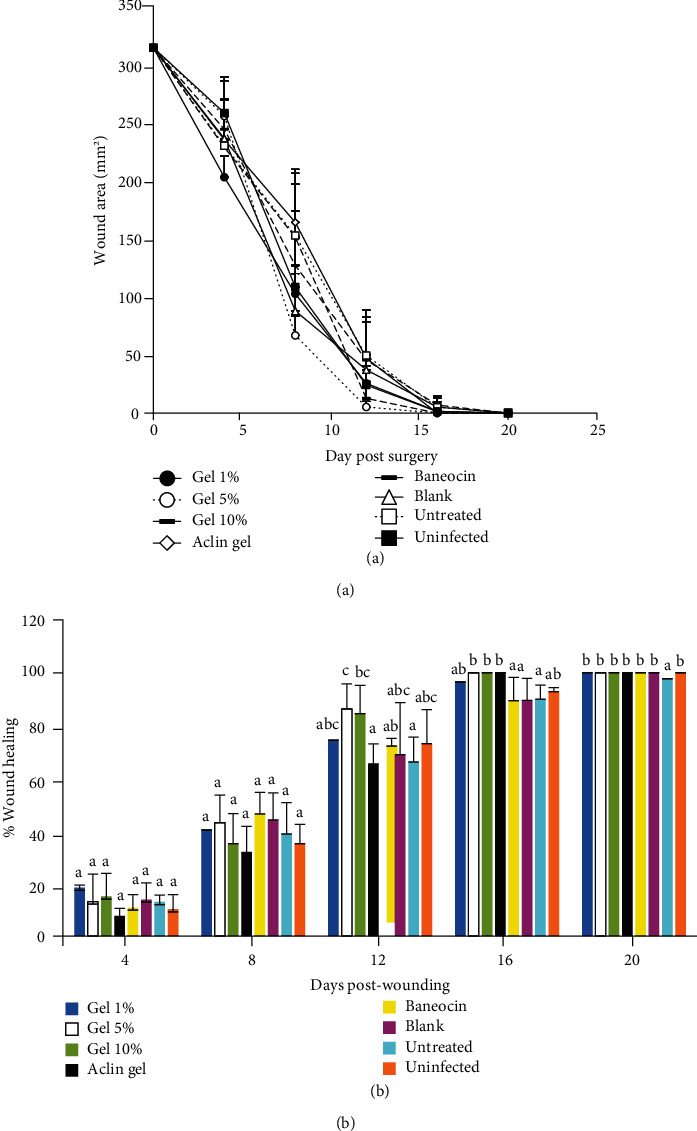
Effect of *C. annuum* fruit extract gels on the wound area (a) and the percentage of wound contraction (b) in excision wound model on different days after wounding. The data represent the mean ± standard deviation; on the same day, the values earmarked with different superscript letters (a–c) are significantly different at *p* < 0.05.

The therapeutic effect of *C. annuum* fruit extract gel on an excision wound infected with *S. aureus* varied with the concentrations and duration of treatment (Figure 7). The percentage of wound contraction increased with the days of treatment. The gel-based extracts showed very close contraction rates on days 4 and 8 regardless of the extract concentration. Meanwhile, on the 16^th^ day, groups 2 and 3 which received 5% and 10% extract gels, respectively, ended up having a contraction rate of 100%. This result is comparable to that of group 4 which received the standard gel (Aclin gel). In contrast, it was observed that the infected wounds treated with 1% extract gel had higher contraction rates than the standard gel (Baneocin) after 16 days though not significantly different (Table 3). Hence, complete healing (close to 100%) was observed in the groups treated with standard, 1%, 5%, and 10% extract gels on day 20.

The results also show that treatment significantly (*p* < 0.05) reduced the number of colony-forming units (CFU) of *S. aureus* at the infection site (Table 3). Interestingly, no CFU of *S. aureus* was observed at the infection site after 20 days of treatment with 5 and 10% of gel based on *C. annuum* fruit extract, clindamycin, and Baneocin. The number of CFU of *S. aureus* at the infection site in the group treated with 1% of gel based on *C. annuum* fruit extract was significantly (*p* < 0.05) lower than those obtained in the untreated group and group treated with 1% Carbopol. These results suggest that 5% and 10% of gel based on *C. annuum* fruit extract may be used to treat wound infected with *S. aureus*. Gel without fruit extract (blank treatment) had a number of CFU of bacteria at the infection site comparable to that of the untreated group.

The serum content in total proteins on day 0 (before starting the treatment) was higher in noninjured rats (5.92 ± 0.24) compared to the tested groups (Table 4). Meanwhile, on day 20, an inverse dose-dependent increase was recorded among the tested groups. Rats treated with 1% gel showed the highest total protein content followed by those receiving 5% and 10% gels. Animals treated with the standard gel (Aclin gel) showed the highest total protein content 6.23 ± 0.49, and this protein content was significantly higher (*p* < 0.05) than that of animals treated with Baneocin ointment. Furthermore, LDH enzymatic activity was elevated in all groups treated with *C. annum* extract gel and this enzymatic activity was significantly different from noninjured rats. Rats treated with Baneocin ointment showed the lowest level of LDH activity compared to noninjured rats.

### 3.4. Toxicological Effect of the MeOH Extract of *C. annuum* Fruits on the Skin and Eye

The irritating effect of gel based on the MeOH extract of *C. annuum* fruits at different therapeutic concentrations on the skin and eye was assessed through skin and eye irritation on the experimental rats. The application of gel-based extract at different concentrations showed no signs of toxicity (no irritation, no edema, and no erythema) on the skin after 72 hours of exposure. Also, the application of the gel-based extract on the eyeball revealed no ocular irritation after 48 hours following clinical observation of the conjunctiva for redness; iris for evaluation of photomotor reflex of the pupil; cornea for the degree of opacity, ulceration, and granulation; and lastly, chemosis for tearing and swelling of the eyelids.

### 3.5. Organoleptic Evaluation

Regardless of concentrations, the gel based on *C. annuum* fruit extract was stable, homogeneous to the naked eye, odorless, orange color ranging from light to dark, thick in consistency, smooth to the touch, and free from air bubbles and lumps over storage time (17 days). The smell was characteristic of chili. After 17 days, the 1% gel began to show signs of fermentation with air bubbles and gas escaping with each opening. Whereas over time (28 days), the organoleptic characteristics of the 5% and 10% gels have remained constant. The 5% and 10% gels therefore exhibited macroscopic stability regardless of the storage temperature (25°C).

### 3.6. Physicochemical Evaluation

#### 3.6.1. pH of the Gel Based on *C. annuum* Fruit Extract

The pH values of the 5% and 10% gels were 6.24 and 5.69 at 37°C, respectively, 24 h after preparing the gels. These pH values remained constant over storage time (28 days). In contrast, 24 h after preparation of the gels, the pH of the 1% gel was 6.79 and decreased over time as follows: 4.83, 3.71, and 3.22 at 37°C on days 18, 23, and 28, respectively.

### 3.7. Chemical Analysis of the MeOH Extract of *C. annuum* Fruits

The chemical investigation of MECa revealed the presence of alkaloids, polyphenols, flavonoids, anthocyanins, anthraquinones, tannins, triterpenes, and saponins while steroids were absent. MECa also contained significant amounts of total phenols, tannins, and flavonoids (Table 5). The analysis of the results also revealed that tannins and flavonoids represented 9.62% and 6.83% of the total phenol content, respectively.

## 4. Discussion

The findings of the present investigation revealed that MECa exhibited different antibacterial activities against pathogenic phenotypes among which the multidrug-resistant bacteria. This result corroborates those of early reports [39–41]. The differences in inhibitory activity observed against the bacterial species would be due to the difference in genetic and structural composition of the isolates and strains. The presence of potential antimicrobial substances was identified by chemical analysis of *C. annuum* fruit extract which showed the presence of alkaloids, polyphenols, flavonoids, anthocyanins, anthraquinones, tannins, triterpenes, and saponins. This partially corroborates the results of previous reports [39, 42] which had demonstrated the presence of some of these groups of bioactive compounds in MECa. The difference in the chemical composition of MECa with previous findings may be due to the variety of the plant tested, solvent of extraction, geographical situation, or the period of harvesting. Depending on the mechanisms of action, the active ingredients could have a bacteriostatic or bactericidal effect on microorganisms. Two studies [8, 9] showed that the substances were considered bacteriostatic agents when the ratio MBC/MIC > 4 and as bactericidal agents when the MBC/MIC ratio ≤ 4. In this study, the methanol fruit extract of *C. annuum* was bactericidal on *S. aureus* 56, *S. aureus* 18, *S. aureus* ATCC 25923, *E. coli* ATCC 8739, and *P. aeruginosa* PA01 whereas it was bacteriostatic on *E. coli* 64R and *E. coli* 141R.

The global burden of antibiotic resistance has revived the interest in evaluating the antimicrobial properties of plants [43]. The antibacterial mechanisms of medicinal plants against microorganisms are known to occur via inhibition of cell wall synthesis [44] and cellular accumulation in the bacteria causing energy depletion [45]. Bacterial cytoplasmic membrane provides barrier to the passage of materials into the protoplasm. This permeability barrier role of cell membranes assists in solute transport, regulation of metabolism, and control of the turgor pressure [46]. In addition, measurements of bacterial membrane permeability are fundamental to study the mechanisms of antimicrobial actions of active principles [47]. In this study, the combination of erythromycin + extract caused a significant (*p* < 0.5) concentration-dependent decrease in the growth of *P. aeruginosa* compared to that of erythromycin alone, suggesting the potentiating effect of MECa on the membrane permeability to erythromycin. Meanwhile, the effect of the *C. annuum* fruit extract alone was lower on the outer membrane permeability of *P. aeruginosa* PA01 and *E. coli* 64R compared with that of *S. aureus* 18. This finding is in agreement with the observation of Vaara [48] who showed that Gram-negative bacteria are more resistant to antibiotics because of a selective barrier possession, represented by the outer membrane of the bacterial cell wall.

This study also showed that the methanol extract of MECa induced significant increases of biological materials absorbed at 260 and 280 nm, suggesting that nucleic acids and proteins were lost through a damaged cytoplasmic membrane. This observation further suggests the contribution of the *C. annuum* fruit extract to the alteration of the microbial membrane, and the resulting leakage of intracellular materials may lead to microbial death, justifying its bactericidal effect. The ability of MECa to alter the bacterial cell membrane was further demonstrated by releasing reducing sugars in the culture suspensions of *P. aeruginosa* PA01, *S. aureus* 18, and *E. coli* 64R. In fact, marked leakage of cytoplasmic materials such as sugar, proteins, and nucleic acids is considered indicative of gross and irreversible damage of the cytoplasmic membrane [49].

Proton pumps constitute a target of choice for antimicrobial compounds [50]. Our results showed that the methanol extract of *C. annuum* fruits inhibited the ATPases/H^+^ proton pumps of the bacteria, indicating that the ATPase/H^+^ proton pumps were potential targets of the *C. annuum* fruit extract. The inhibition of these pumps by MECa will therefore be deleterious for bacteria because it will prevent the excretion of protons in the external environment, thus making the environment less acidic, compromising the survival of the bacteria. This inhibition also confirms the bacteriostatic and bactericidal activities observed above.

The enzymatic activity of the respiratory chain dehydrogenases in the bacterial suspensions was inhibited by the *C. annuum* fruit extract. This result suggests that the active components of MECa might bind to the cell surface and then penetrate to the target sites, possibly the phospholipid bilayer of the cytoplasmic membrane and also membrane-bound enzymes. These effects might include the inhibition of proton motive force, inhibition of substrate oxidation, and inhibition of the respiratory chain components and electron transfer [51].

Biofilm development is a mechanism of resistance developed by bacteria to resist the action of drugs. Some plant extracts express their bactericidal activities by the inhibition of the biofilm formation. The results of the present study revealed the capacity of MECa to inhibit 53.8, 53.4%, and 35.34% of the formation of biofilms in *S. aureus* 18, *P. aeruginosa* PA01, and *E. coli* 64R, respectively. This ability to inhibit the formation of biofilms resides in the rather rich and diverse phytochemical composition of the extract. In fact, plant extract components inhibit peptidoglycan synthesis and modulate the quorum sensing of a whole gene intervening in the regulation of biofilm formation [51]. Intriguingly, this is the first report on the antibacterial mechanisms of action of MECa.

The gel based on *C. annuum* fruit extract showed a significant increase in the percentage of wound closure and caused a significant reduction of the number of colony-forming units (CFU) of *S. aureus* at the infection site. This result suggests that *C. annuum* fruit extract enhanced wound healing and has antibacterial properties. In fact, wound contraction accounts for 88% of the healing process [52] whereas infection of a wound can seriously delay the healing process by causing the formation of poor-quality granulation tissue, reducing the tensile strength of the connective tissue as well as the loss of epithelization and the appearance of odor [53, 54]. The total phenols, flavonoids, and tannins found in the *C. annuum* fruit extract have been shown to be important for wound healing [55]. Indeed, flavonoids are free radical species scavenger and reported to play an important role in accelerating the wound-healing process [56] and tannins to hasten the healing of wounds and inflamed mucus membrane [57]. This means that both metabolite groups are responsible for the contraction of the wound and the increase in the rate of epithelialization [58]. To the best of our knowledge, this is a pioneer report demonstrating the therapeutic effect of MECa against infected wound in a rat model with its mechanisms of antibacterial action.

The findings of the present study also showed that the extract gels and Aclin gel recorded the highest total protein content of granulation tissues as compared to the untreated control groups. In fact, tissue proteins like collagen help strengthen and support cell tissue and are used as biochemical markers, indicating better healing quality medication in wounds [59, 60]. The activities of LDH were significantly higher in wounded rats compared to unwounded rats. Elevated serum LDH levels have been reported as diagnostic biomarkers for various diseases in humans, including the life-threatening bacterial infections [61]. A previous study had shown that serum LDH levels were higher in rats infected with *Francisella tularensis* and *Salmonella enterica* serovar Typhimurium than those obtained in uninfected rats and would be related directly to the size of the infecting dose and the severity of the response produced [62]. Although the mode of inoculation was topical, we think that an elevated level of LDH was due to the dissemination of *S. aureus* through the interstitial space into internal tissues where it could have caused tissue damage that led to high levels of LDH. A clinical test is required to confirm this result. Finally, toxicological tests showed that MECa is nonirritating to the skin (primary irritation index of the gel, PII = 0) and slightly irritating to the eyes (average eye irritation index, AEII = 0) [33] and therefore should be used with caution.

The pH values of the gels were 6.79, 6.24, and 5.69 for 1%, 5%, and 10% (*w*/*w*) of gel based on *C. annuum* fruit extract at 37°C, respectively. These pH values correspond to the pH of cosmetic formulas which is generally between 5 and 7. These preparations are therefore suitable for skin application since they are compatible with the pH of the skin between 5 and 7 [63]. Regarding the pH of the 1% gel based on *C. annuum* fruit extract, signs of the fermentation with air bubbles and gas escaping with each opening were observed after 17 days of storage, indicating that the storage conditions influence the pH stability of the 1% gel based on *C. annuum* fruit extract. However, no change was observed during the days of storage for the 5% and 10% gels based on *C. annuum* fruit extract. The storage conditions therefore did not influence the pH stability of these formulations, which remained compatible with that of the skin [63]. On the other hand, pH stability over time is a good sign of microbial nonproliferation. In fact, an early report has shown that the stability of the pH over time is a marker which could be an indicator of nonmicrobial contamination [63].

This study allowed us to identify the formulations of 5% and 10% gels based on *C. annuum* fruit extract which were stable, odorless, orange color ranging from light to dark, smooth to the touch, and homogeneous to the naked eye. During 28 days, evaluations including organoleptic controls and physicochemical control showed that the 5% and 10% gels based on *C. annuum* fruit extract remained stable.

## 5. Conclusion

The present study demonstrates the antibacterial and therapeutic properties of the *C. annuum* fruit extract in an infected wound in a rat model. The groups of bioactive compounds found in this extract act by inhibiting biofilm formation, ATPases/H^+^ proton pumps, and dehydrogenase activity as well as by alteration of the bacterial cell membrane through the leakage of nucleic acids, reducing sugars and total proteins. The results of this study support the traditional use of *C. annuum* fruits in the treatment of wounds and infectious diseases.

## Figures and Tables

**Table 1:** Characteristics of bacteria used in the study.

Bacteria	Features	Reference
*S. aureus*		
ATCC 25923	Reference strain	/
*S. aureus* 56	Clinical isolate: AMP^r^, DOX^r^	[64]
*S. aureus* 18	Clinical isolate: AMP^r^, DOX^r^, VAN^r^	[64]
*E. coli*		
ATCC 8739	Reference strain	[65]
*E. coli* 64R	Clinical isolate: CTR^r^, NIT^s^, GEN^r^, CHL^r^, CPD^r^, DOX^r^, NOR^r^, ERY^r^, COT^r^, CAZ^r^	[66]
*E. coli* 141R	Clinical isolate: CTR^r^, NIT^s^, GEN^s^, CHL^r^, CPD^r^, DOX^r^, NOX^r^, ERY^r^, COT^r^, CAZ^s^	[66]
*P. aeruginosa*		
PA01	Reference strain	[67]

AMP: ampicillin; VAN: vancomycin; DOX: doxycycline; CTR: ceftriaxone; NIT: nitrofurantoin; GEN: gentamicin; CHL: chloramphenicol; CPD: cefpodoxime; NOR: norfloxacin; ERY: erythromycin; CAZ: ceftazidime; COT: cotrimoxazole; r: resistant; s: sensitive.

**Table 2:** Antibacterial activity (MIC and MBC in *μ*g/mL) of the MeOH extract of *C. annuum* fruits against Gram-positive and Gram-negative bacteria.

Bacteria	MeOH extract of *Capsicum annuum* fruits		Amoxicillin trihydrate		Cefotaxime		Levofloxacin hemihydrate		Erythromycin	
	MIC	MBC	MIC	MBC	MIC	MBC	MIC	MBC	MIC	MBC
*S. aureus* 56	256	1024	0.25	1	2	8	0.06	0.25	2	16
*S. aureus* 18	64	256	64	128	16	64	16	64	16	64
*S. aureus* ATCC 25923	256	512	16	64	32	128	0.25	0.5	0.03	0.06
*E. coli* ATCC 8739	256	256	1024	/	64	64	0.5	1	256	/
*E. coli* 64R	2048	/	1024	/	16	256	0.5	1	64	128
*E. coli* 141R	512	/	4	32	32	128	0.25	1	0.5	4
*P. aeruginosa* PA01	256	1024	16	64	32	128	0.12	0.25	0.12	8

MIC: minimum inhibitory concentration; MBC: minimum bactericidal concentration; /: >2048 *μ*g/mL.

**Table 3:** Effect of *C. annuum* fruit extract gels on *S. aureus* load (CFU/mg) of wounds after 20 days of treatment.

Treatment	Day 0	Day 20
Gel 1%	700000 ± 17248.19^b^	2000 ± 1224.74^c^
Gel 5%	742000 ± 22616.37^b^	0 ± 0^a^
Gel 10%	738000 ± 17000^b^	0 ± 0^a^
Aclin gel	775000 ± 19937.4^b^	0 ± 0^a^
Baneocin	750000 ± 15165.75^b^	0 ± 0^a^
Blank	876000 ± 113791.5^b^	35000 ± 7416.19^d^
Untreated	891000 ± 85839.97^b^	50000 ± 16507.57^d^
Uninfected	0 ± 0^a^	0 ± 0^a^

The data represent the mean ± standard error; on the same column, the values earmarked by different superscript letters (a–d) are significantly different at *p* < 0.05.

**Table 4:** Serum levels of total proteins and enzymatic activity of LDH in experimental animals after 20 days of treatment.

Treatments	Total proteins (g/dL)		LDH (IU/L)	
	Day 0	Day 20	Day 0	Day 20
Gel 1%	5.06 ± 0.28^abc^	5.94 ± 0.24^c^	251.95 ± 31.36^a^	546.09 ± 190.97^abc^
Gel 5%	4.90 ± 0.62^abc^	5.2 ± 1.36^abc^	239.81 ± 81.96^a^	852.68 ± 199.48^bc^
Gel 10%	5.82 ± 0.58^bc^	5.02 ± 1.28^abc^	432.07 ± 53.62^b^	658.48 ± 124.95^abc^
Aclin gel	4.50 ± 0.58^ab^	6.23 ± 0.49^c^	176.06 ± 10.11^a^	448.55 ± 85.01^ab^
Baneocin	5.26 ± 0.66^abc^	4.3967 ± 0.78^ab^	203.38 ± 3.03^a^	274.72 ± 41.86^a^
Blank	4.03 ± 0.23^a^	4.4695 ± 0.47^bc^	260.05 ± 65.77^a^	622.26 ± 108.01^abc^
Untreated	4.50 ± 0.03^bc^	3.81 ± 1.19^a^	424.48 ± 32.88^b^	901.88 ± 99.05^c^
Uninfected	4.12 ± 0.43^a^	4.03 ± 1.14^ab^	310.39 ± 27.06^ab^	492.17 ± 79.69^abc^
Unwounded	5.92 ± 0.24^c^	5.38 ± 0.63^bc^	259.37 ± 24.89^a^	284.27 ± 46.80^a^

The data represent the mean ± standard error; on the same column, the values earmarked by different superscript letters (a–c) are significantly different at *p* < 0.05; LDH: lactate dehydrogenase.

**Table 5:** Total phenol, flavonoid, and tannin contents of the MeOH extract of *C. annuum* fruits.

Sample	Total flavonoids (*μ*g ECAT/mg of extract)	Total phenols (*μ*g EGA/mg of extract)	Tannins (*μ*g ETA/mg)
*C. annuum* fruit extract	7.23 ± 1.5^a^	105.8 ± 2.71^c^	10.18 ± 0.92^b^

Data are expressed by mean ± standard deviation (SD). Values affected by the different superscript letters (a–c) are significantly different (*p* < 0.05).

## Data Availability

The datasets generated and analysed during the current study are available from the corresponding author upon reasonable request.

## Supplementary Materials

Supplementary Materials Figure S1: macroscopic changes in the skin wound sites in rats treated with C. *annuum* fruit extract gels on days 0, 8, and 20 after wounding.

## References

[1] Rakhimov K. D., Pak R. N., Tekhneryadnov A. V. (2000). Wound healing activity of Bialm ointment. *Pharmaceutical Chemistry Journal*.

[2] Gupta A., Kumar R., Pal K., Singh V., Banerjee P. K., Sawhney R. C. (2006). Influence of sea buckthorn (*Hippophae rhamnoides* L.) flavone on dermal wound healing in rats. *Molecular and Cellular Biochemistry*.

[3] Priya K. S., Gnanamani A., Radhakrishnan N., Babu M. (2002). Healing potential of _Datura alba_ on burn wounds in albino rats. *Journal of Ethnopharmacology*.

[4] Hollinworth H. (1997). The management of infected wounds. *Professional Nurse*.

[5] Muhammad H. S., Muhammad S. (2005). The use of *Lawsonia inermis* Linn. (henna) in the management of burn wound infections. *African Journal of Biotechnology*.

[6] Buru A. S., Pichika M. R., Neela V., Mohandas K. (2014). _In vitro_ antibacterial effects of _Cinnamomum_ extracts on common bacteria found in wound infections with emphasis on methicillin-resistant _Staphylococcus aureus_. *Journal of Ethnopharmacology*.

[7] Hancock R. E. (2005). Mechanisms of action of newer antibiotics for Gram-positive pathogens. *Lancet Infectious Diseases*.

[8] Tamokou J. D. D., Tala F. M., Wabo K. H., Kuiate J. R., Tane P. (2009). Antimicrobial activities of methanol extract and compounds from stem bark of _Vismia rubescens_. *Journal of Ethnopharmacology*.

[9] Djouossi M. G., Tamokou J. D. D., Ngnokam D. (2015). Antimicrobial and antioxidant flavonoids from the leaves of *Oncoba spinosa* Forssk (Salicaceae). *BMC Complementary and Alternative Medicine*.

[10] Mabou F. D., Tamokou J. D. D., Ngnokam D., Voutquenne-Nazabadioko L., Kuiate J. R., Bag P. K. (2016). Complex secondary metabolites from *Ludwigia leptocarpa* with potent antibacterial and antioxidant activities. *Drug Discoveries and Therapeutics*.

[11] Nzogong T. R., Ndjateu F. S. T., Ekom S. E. (2018). Antimicrobial and antioxidant activities of triterpenoid and phenolic derivatives from two Cameroonian Melastomataceae plants: *Dissotis senegambiensis* and *Amphiblemma monticola*. *BMC Complementary and Alternative Medicine*.

[12] Ortega M. H., Moreno A. O., Navarr M. D. H., Cevallos G. C., Alvarez L. D., Mondragon H. N. (2012). Antioxidant, antinociceptive, and anti-inflammatory effects of carotenoids extracted from dried pepper (*Capsicum annuum* L.). *Journal of Biomedicine and Biotechnology*.

[13] Ramchiary N., Kehie M., Brahma V., Kumaria S., Tandon P. (2014). Application of genetics and genomics towards *Capsicum* translational research. *Plant Biotechnology Reports*.

[14] Kumar O. A., Tata S. S. (2009). Ascorbic acid contents in Chili peppers (Capsicum L.). *Notulae Scientia Biologicae*.

[15] Hill T. A., Ashrafi H., Reyes-Chin-Wo S. (2013). Characterization of *Capsicum annuum* genetic diversity and population structure based on parallel polymorphism discovery with a 30K unigene Pepper GeneChip. *PLoS One*.

[16] Oyedemi B. O., Kotsia E. M., Stapleton P. D., Gibbons S. (2019). Capsaicin and gingerol analogues inhibit the growth of efflux-multidrug resistant bacteria and R-plasmids conjugal transfer. *Journal of Ethnopharmacology*.

[17] Füchtbauer S., Mousavi S., Bereswill S., Heimesaat M. M. (2021). Antibacterial properties of capsaicin and its derivatives and their potential to fight antibiotic resistance – a literature survey. *European Journal of Microbiology and Immunology*.

[18] Ekom S. E., Tamokou J. D. D. (2018). Methanol leaves extract of Psidium guajava Linn. exhibited antibacterial and wound healing activities. *International Journal of Current Microbiology and Applied Sciences*.

[19] Nguemeving J. R., Azebaze A. G., Kuete V. (2006). Laurentixanthones A and B, antimicrobial xanthones from _Vismia laurentii_. *Phytochemistry*.

[20] Nono E. C., Mkounga P., Kuete V., Marat K., Hultin P. G., Nkengfack A. E. (2010). Pycnanthulignenes A-D, antimicrobial cyclolignene derivatives from the roots of Pycnanthus angolensis. *Journal of Natural Products*.

[21] CLSI (1997). *Methods for dilution antimicrobial susceptibility tests for bacteria that grow aerobically. Approved Standards, M7A4*.

[22] CLSI (1999). *Methods for determining bactericidal activity of antimicrobial agents. Approved guideline, M26-A*.

[23] Oliveira D. M., Melo F. G., Balogun S. O. (2015). Antibacterial mode of action of the hydroethanolic extract of _*Leonotis nepetifolia*_ (L.) R. Br. involves bacterial membrane perturbations. *Journal of Ethnopharmacology*.

[24] Miller G. (1959). Use of Dinitrosalicylic acid reagent for determination of reducing Sugar. *Analytical Chemistry*.

[25] Bradford M. (1976). A rapid and sensitive method for the quantitation of microgram quantities of protein utilizing the principle of protein-dye binding. *Analytical Biochemistry*.

[26] Kim K. J., Sung W. S., Suh B. K. (2009). Antifungal activity and mode of action of silver nanoparticles on *Candida albicans*. *Biometals*.

[27] Li W. R., Xie X. B., Shi Q. S., Zeng H. Y., OU-Yang Y. S., Chen Y. B. (2010). Antibacterial activity and mechanism of silver nanoparticles on *Escherichia coli*. *Applied Microbiology and Biotechnology*.

[28] Manavathu E. K., Dimmock J. R., Sarvesh C. V., Chandrasekar P. H. (2001). Inhibition of H+-ATPase mediated proton pumping in *Cryptococcus neoformans* by a novel conjugated styryl ketone. *Journal of Antimicrobial Chemotherapy*.

[29] Nikolic M., Vasic S., Djurdjevic J., Stefanovic O., Comic L. (2014). Antibacterial and anti-biofilm activity of ginger *(Zingiber officinale* (Roscoe)) ethanolic extract. *Kragujevac Journal of Science*.

[30] Okoli C. O., Ezike A. C., Akah P. A. (2009). Studies on wound healing and antiulcer activities of extract of aerial parts of *Phyllanthus niruri* L. (Euphorbiaceae). *American Journal of Pharmacology and Toxicology*.

[31] Luepke N. P. (1986). The Hen’s egg test (HET): an alternative toxicity test. *British Journal of Dermatology*.

[32] OECD (1987). Acute dermal toxicity. *Guidelines for Testing of Chemicals*.

[33] OECD *Test No 405. Acute eye irritation/corrosion, OECD guideline for the testing of chemicals*.

[34] Brossard D., Chaumeil J. C., Le H. I. R. (2016). *Pharmacie Galénique: Bonne pratique de fabrication des médicaments*.

[35] Trease G. E., Evans W. C. (1989). *A Text Book of Pharmacognosy*.

[36] Padmaja M., Sravanthi M., Hemalatha K. P. J. (2011). Evaluation of antioxidant activity of two Indian medicinal plants. *Journal of Phytology*.

[37] Govindappa M., Channabasava R., Sowmya D. V. (2011). Phytochemical Screening, Antimicrobial and _in vitro_ Anti-inflammatory Activity of Endophytic Extracts from _Loranthus_ sp.. *Journal of Pharmacognosy and Phytochemistry*.

[38] Ramde-Tiendrebeogo A., Tibiri A., Hilou A. (2012). Antioxidative and antibacterial activities of phenolic compounds from *Ficus sur Forssk.* and *Ficus sycomorus* L. (Moraceae) : potential for sickle cell disease treatment in Burkina Faso. *International Journal of Biological and Chemical Sciences*.

[39] Koffi-Nevry R., Kouassi K. C., Nanga Z. Y., Koussémon M., Loukou G. Y. (2012). Antibacterial activity of two bell pepper Extracts:Capsicum *annuumL. andCapsicum frutescens*. *International Journal of Food Properties*.

[40] Oulaï A. C., Djè K. M., Eba K. P., Adima A. A., Kouadio E. J. P. (2018). Chemical composition, antioxidant and antimicrobial activities of *Capsicum annuum* var. annuum concentrated extract obtained by reverse osmosis. *GSC Biological and Pharmaceutical Sciences*.

[41] Anikwe L. U., Onoja U. S., Onyeke C. C., Nweze E. I. (2017). Antimicrobial activities of four varieties of *Capsicum annuum* fruits cultivated in Southeast Nigeria against multidrug-resistant and susceptible organisms. *Journal of Basic Pharmacology and Toxicology*.

[42] Samrot V. A., Shobana N., Jenna R. (2018). Antibacterial and antioxidant activity of different staged ripened fruit of *Capsicum annuum* and its green synthesized silver nanoparticles. *BioNanoScience*.

[43] Hyldgaard M., Mygind T., Meyer R. L. (2012). Essential oils in food preservation: mode of action, synergies, and interactions with food matrix components. *Frontiers in Microbiology*.

[44] Cowan M. M. (1999). Plant products as antimicrobial agents. *Clinical Microbiology Reviews*.

[45] Conner D. E., Davidson P. T., Branene A. I. (1993). Naturally occurring compounds. *Antimicrobials in foods*.

[46] Cox S. D., Mann C. M., Markham J. L. (2000). The mode of antimicrobial action of the essential oil of *Melaleuca alternifolia (tea tree oil)*. *Journal of Applied Microbiology*.

[47] Ohmizo C., Yata M., Katsu T. (2004). Bacterial cytoplasmic membrane permeability assay using ion-selective electrodes. *Journal of Microbiological Methods*.

[48] Vaara M. (1992). Agents that increase the permeability of the outer membrane. *Microbiological Reviews*.

[49] Krishnan R., Murugan K. (2016). Bactericidal potentiality of flavonoids extracted from cell suspension cultures of *Marchantia linearis* Lehm & Lindenb. *International Journal of Pharmacy*.

[50] Silva N. C. C., Fernandes Júnior A. (2010). Biological properties of medicinal plants: a review of their antimicrobial activity. *Journal of Venomous Animals and Toxins Including Tropical Diseases*.

[51] Lu C., Kirsch B., Zimmer C. (2012). Discovery of Antagonists of PqsR, a Key Player in 2-Alkyl-4-quinolone- Dependent _Quorum Sensing_ in _Pseudomonas aeruginosa_. *Chemistry and Biology*.

[52] Ejaz S., Chekarova I., Cho J. W., Lee S. Y., Ashraf S., Lim C. W. (2009). Effect of aged garlic extract on wound healing: a new frontier in wound management. *Drugs and Chemical Toxicology*.

[53] Ibrahim N., Wong S., Mohamed I. (2011). Wound healing properties of selected natural products. *International Journal of Environmental Research and Public Health*.

[54] Annan K., Houghton P. J. (2008). Antibacterial, antioxidant and fibroblast growth stimulation of aqueous extracts of _Ficus asperifolia_ Miq. and _Gossypium arboreum_ L., wound- healing plants of Ghana. *Journal of Ethnopharmacology*.

[55] Mulisa E., Asres K., Engidawork E. (2015). Evaluation of wound healing and anti-inflammatory activity of the rhizomes of *Rumex abyssinicus* J. (Polygonaceae) in mice. *BMC Complementary and Alternative Medicine*.

[56] Kim H., Kawazoe T., Han D. W. (2008). Enhanced wound healing by an epigallocatechin gallate-incorporated collagen sponge in diabetic mice. *Wound Repair and Regeneration*.

[57] Okwu D. E., Okwu M. E. (2004). Chemical composition of *Spondias mombia* Linn plant parts. *Journal of Sustainable Agriculture and Environment*.

[58] Ekom S. E., Tamokou J. D. D., Kuete V. (2022). Methanol extract from the seeds of _Persea americana_ displays antibacterial and wound healing activities in rat model. *Journal of Ethnopharmacology*.

[59] Tang T., Yin L., Yang J., Shan G. (2007). Emodin, an anthraquinone derivative from _Rheum officinale_ Baill, enhances cutaneous wound healing in rats. *European Journal of Pharmacology*.

[60] Paschapur M. S., Patil M. B., Kumar R., Patil S. R. (2009). Evaluation of aqueous extract of leaves of *Ocimum kilimandscharicum* on wound healing activity in albino *Wistar* rats. *International Journal of Pharm Tech Research*.

[61] Balasubramanian S., Kaarthigeyan K., Srinivas S., Rajeswari R. (2010). Serum ALT: LDH ratio in typhoid fever and acute viral hepatitis. *Indian Pediatrics*.

[62] Woodward J. M., Camblin M. L., Jobe M. H. (1969). Influence of bacterial infection on serum enzymes of white rats. *Applied Microbiology*.

[63] Rosso L., Lobry J.-R., Bajard S., Flandrois J.-P. (1995). Convenient model to describe the combined effects of temperature and pH on microbial growth. *Applied and Envirommental Microbiology*.

[64] Ngalani O. J., Marbou J. W., Mbaveng A. T., Kuete V. (2020). Immunological profile and bacterial drug resistance in pregnant women: a cross sectional study. *Osong Public Health and Research Perspectives*.

[65] Kuete V., Ngameni B., Tangmouo J. G. (2010). Efflux pumps are involved in the Defense of gram-negative Bacteria against the natural products Isobavachalcone and Diospyrone. *Antimicrobial Agents and Chemotherapy*.

[66] Marbou W. J. T., Jain P., Samajpati S. (2020). Profiling virulence and antimicrobial resistance markers of enterovirulent *&lt;italic&gt;Escherichia Coli&lt;/italic&gt;* from fecal isolates of adult patients with enteric infections in West Cameroon. *Osong Public Health and Research Perspectives*.

[67] Lorenzi V., Muselli A., Bernardini A. F. (2009). Geraniol restores antibiotic activities against multidrug-resistant isolates from gram-negative species. *Antimicrobial Agents and Chemotherapy*.

