# Supplementary material for: Antibacterial and Therapeutic Potentials of the Capsicum annuum Extract against Infected Wound in a Rat Model with Its Mechanisms of Antibacterial Action
Source: Biomed Res Int. 2021 Oct 4;2021:4303902. doi: 10.1155/2021/4303902 (PMC8505066; doi:10.1155/2021/4303902)
Supplement: Supplementary Materials — Figure S1: macroscopic changes in the skin wound sites in rats treated with C. annuum fruit extract gels on days 0, 8, and 20 after wounding. [file 4303902.f1.pdf]

Antibacterial and therapeutic potentials of the *Capsicum annuum* extract against infected wound in rat model with its mechanisms of antibacterial action

Steve Endeguele Ekom, Jean-De-Dieu Tamokou* and Victor Kuete

Supplementary material

List of Contents

Cover page 1

FIGURE S1: Macroscopic changes in the skin wound sites in rats treated with *C. annuum* fruit extract gels on days 0, 8 and 20 after wounding 3

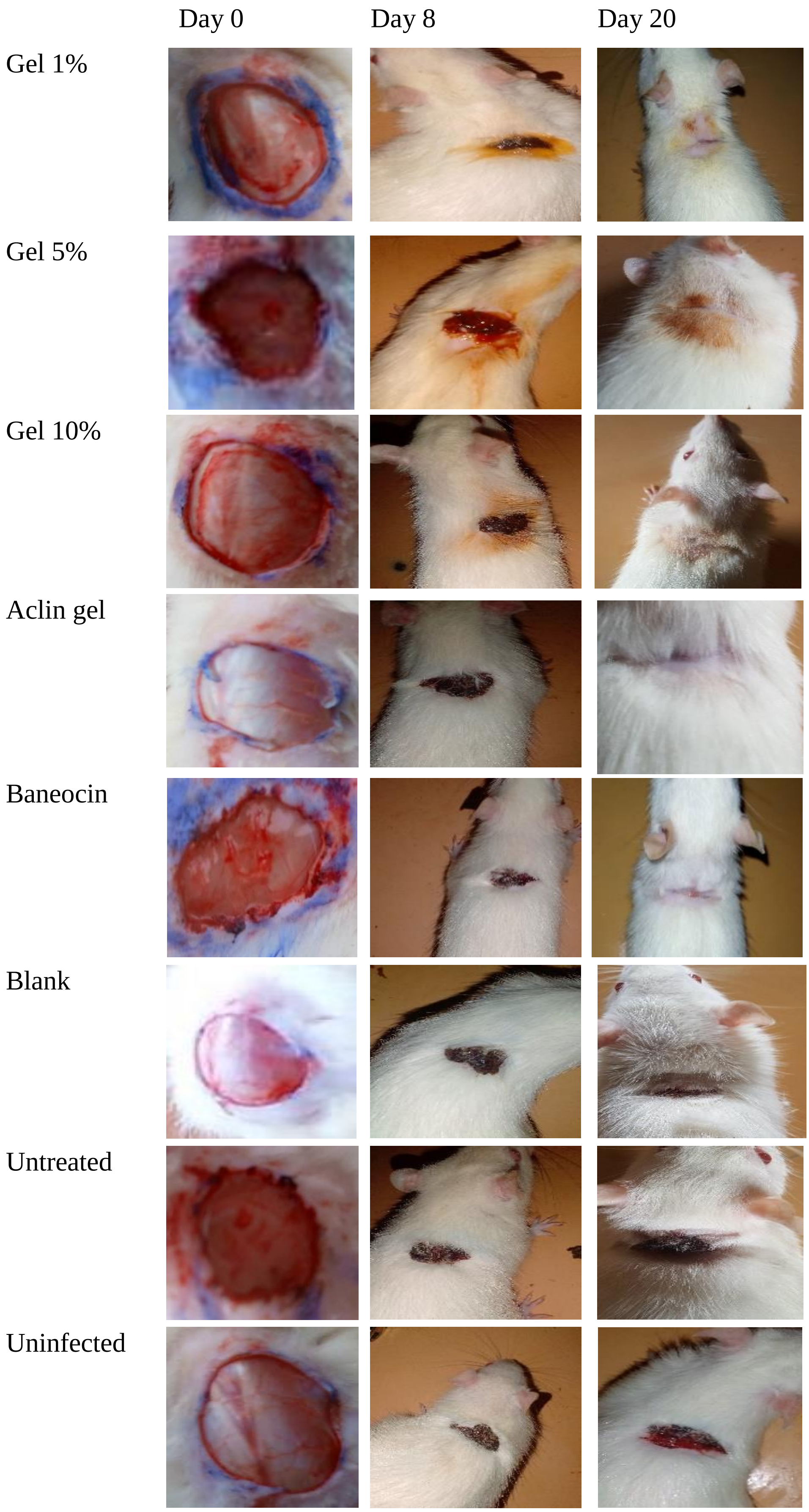

FIGURE S1: Macroscopic changes in the skin wound sites in rats treated with *C. annuum* fruit extract gels on days 0, 8 and 20 after wounding.
